# Jump In! An Investigation of School Physical Activity Climate, and a Pilot Study Assessing the Acceptability and Feasibility of a Novel Tool to Increase Activity during Learning

**DOI:** 10.3389/fpubh.2014.00058

**Published:** 2014-05-28

**Authors:** Dan J. Graham, Rachel G. Lucas-Thompson, Maeve B. O’Donnell

**Affiliations:** ^1^Department of Psychology, Colorado State University, Fort Collins, CO, USA; ^2^Department of Human Development and Family Studies, Colorado State University, Fort Collins CO, USA

**Keywords:** physical activity, focus groups, primary school children, health promotion, sitting reduction

## Abstract

Physical activity (PA) benefits children’s physical and mental health and enhances academic performance. However, in many nations, PA time in school is decreasing under competing pressures for time during the school day. The present paper argues that PA should not be reduced or seen as incompatible with academic learning. Instead, the authors contend that it is critical to develop tools that incorporate PA into content learning during the school day. To facilitate the development of such tools, the authors conducted 6 focus group discussions with 12 primary school teachers and administrators to better understand the school climate around PA as well as school readiness to embrace PA tools that can be used during academic content learning. In addition, a pilot test of a new health promotion tool, the *Jump In!* educational response mat, was conducted with 21 second-grade students from one classroom in Northern Colorado in 2013. The results of both studies demonstrated acceptability and feasibility of incorporating PA into classroom learning, and suggested that tools like *Jump In!* may be effective at overcoming many of the PA barriers at schools. Teachers and administrators valued PA, believed that students were not getting enough PA, and were receptive to the idea of incorporating PA into classroom learning. Students who used *Jump In!* mats during a math lesson reported more interest in the class material and rated themselves as more alert during the lesson, compared to students who did not use the response mats. In addition, incorporating PA into the lesson did not impair performance on a quiz that assessed learning of the math content. *Jump In!* mats were successfully integrated into the lesson plan and were well-received by teachers and students. Together, the results of these studies suggest that, given the right tools, incorporating more PA into classroom learning may be beneficial and well-received by students, teachers, and administrators.

## Introduction

The physical health benefits of regular physical activity (PA) for individuals of all ages (e.g., greater quality and length of life, reduced incidence of acute and chronic mental and physical illness) are well-established (1–3). Thus, PA comprises a critical element of health promotion among youth. In addition, PA can also enhance learning (4, 5). Bouts of PA augment cognition (6), and the effects of PA on the brain, induced by increased flow of blood, norepinephrine, and endorphins can “reduce stress, improve mood, induce a calming effect after exercise, and perhaps as a result improve achievement” [Ref. (7), p. 214]. A 2005 review of the effects of PA on health and behavior outcomes for youth reported that a variety of research types have demonstrated links between PA and gains in academic performance, concentration, memory, and classroom behavior (8). Although these benefits have largely been linked with moderate-to-vigorous PA, it has recently become clear that PA of lighter intensity may also produce similar benefits, particularly if the light-intensity PA replaces sitting (9).

These various physical and cognitive benefits provided by PA suggest that PA would be of considerable benefit for all people, certainly including school-aged children and adolescents for whom daily learning is their primary task. In the United States of America (USA), 94–98% of school-aged children and adolescents are enrolled in school for an average of 35 h per week (10). Despite these indications that PA can benefit both student health and academic performance, most children in the USA do not achieve the recommended 60 min of daily PA and PA in schools is declining (11); internationally, many countries have rates of child and adolescent PA that are even lower than in the USA [e.g., Ref. (12)], and rates of in-school PA are also declining internationally (13).

In the USA, physical education (PE) time has been reduced in many schools due to efforts to increase time spent in content-area learning (14), despite recommendations from international working groups that increasing PA at school is a high-leverage target for promoting health worldwide (15). Preparing students to achieve specific standards for content-area courses, but not PA, has meant reduced incentive and thus reduced time for PA at school. In many countries, PA on the way to and from school (i.e., active commuting) has also decreased in recent years [e.g., Ref. (16–22)]. Social and environmental changes have made actively commuting less attractive for many and not possible for some (e.g., due to safety concerns and constraints imposed by the built environment).

As a result, at a time when meeting academic standards is being greatly emphasized, PA in schools is decreasing despite its demonstrated ability to enhance cognitive and physical health outcomes (8). Research demonstrating that PA can benefit learning suggests that removing PA time from the school day to make room for more academic time may not only be harmful to students’ physical health, but may also fail to improve (or actually worsen) academic outcomes. Indeed, spending more, rather than less, time being physically active may provide a greater boost to academic outcomes while also improving student health. In addition, students allowed to move around more during the school day may be better able to focus on learning. A 2010 CDC executive summary of research on school-based PA and academic performance reported that of nine studies that had explored PA in the classroom, eight of the studies suggested that more classroom-based PA was related to more-positive cognitive and academic behaviors and attitudes, and none of the studies suggested that more PA was detrimental to cognition and achievement (23).

Due to the prevailing primary school educational atmosphere in the USA being largely focused on achieving specific educational standards, elements not included among those standards (e.g., PA) may be viewed as non-essential; thus, it is important to devise PA tools that can be used during academic content learning, without taking away time from instruction. To facilitate the development of tools that can be incorporated by teachers into content learning, it is essential to better understand the school climate around PA, as well as school readiness to utilize such tools. Broadly, “school climate” refers to the overall quality of the school environment, and more specifically: the quality of interactions between students, as well as between students and teachers, and the extent to which students have autonomy in decision-making and rules and guidelines are fair and clearly communicated to students (24); school climate is very strongly related to student success and adjustment (24). The school climate construct has recently been applied to PA specifically, and encompasses issues related to adequate facilities, interpersonal relationships that are respectful of physical and social changes that occur during childhood and adolescence, and norms that support PA (25). There is increasing evidence that the school climate around PA is an important predictor of student engagement in PA (25, 26). Because incorporating PA into classroom learning would require a qualitative shift in thinking about PA at school, we considered it necessary to first assess attitudes of teachers and school administrators about PA at school, as well as their openness to incorporating PA into content learning. Therefore, our first step was to conduct a health promotion needs assessment surrounding the current PA climate in primary schools in one school district in Northern Colorado. We aimed to determine: (a) when, where, and what types of PA, teachers currently see occurring in their schools (to identify possible needs and opportunities for PA to be increased), (b) whether teachers and administrators believe that PA is beneficial to students and whether greater rates of PA would benefit students (to determine if school staff have attitudes that would support or hinder increasing PA), (c) what the school-based obstacles to PA are (to understand what barriers new PA tools would need to overcome), and (d) how school staff responded to a health promotion tool that increases PA in the classroom (to understand openness to qualitative shifts in thinking and acting in relation to PA in the classroom). In addition, we conducted a pilot test of a health promotion tool that increases PA during content learning in the classroom, a device called the *Jump In!* educational response mat.

**Figure 1:**
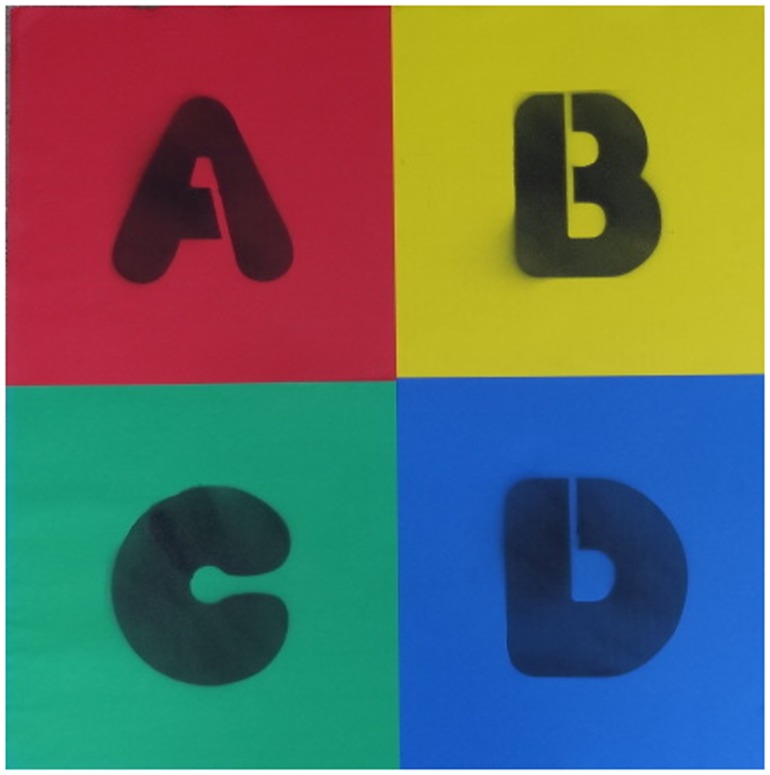
***Jump In*! educational response mat**.

*Jump In!* educational response mats, created by the study team for use in a classroom environment, are 2 × 2 ft mats that can fit behind or next to student desks in a classroom setting. Mats are comprised of four equally sized and differently colored squares lettered “A,” “B,” “C,” and “D” (see Figure 1). The mat design was chosen with several considerations in mind: (1) the presence of four answer choices allows the mats to be used with multiple-choice questions that employ a common number of answer choices (i.e., four); (2) the squares are large enough for children to jump into them with room to spare, so that it is clear which answer choice is being selected; (3) the use of both colors and letters permits mats to be used by even very young children who have learned colors, even before they can read letters.

Students jump on the lettered sections of the *Jump In!* mats to respond to teacher queries (e.g., multiple-choice questions with different letter or color response options), to answer mathematical questions (e.g., answering “what is 10 divided by 2?” by jumping 5 times on the mat), to volunteer answers in class (like a more active hand raise – teachers can ask students to “Jump in when you know the answer,” rather than “Raise your hand when you know the answer”), or for countless other educational purposes. Easy-to-use, durable “Jump In” mats placed on classroom floors allow students to jump to answer questions in class, rather than remaining sedentary during content learning. Mats can incorporate response clicker technology already in place in some classrooms (e.g., iClickers™), but replace handheld response boxes with a floor-based mat format that encourages PA.

The present research is comprised of two studies: Study 1, a health promotion needs assessment that included focus group discussions with school teachers and administrators regarding the current PA climate, school and school personnel readiness to change, and perceived benefits and barriers to increased PA in general as well as with *Jump In!* in particular. Study 2, a process and impact evaluation of a health promotion pilot project that tested the effects on academic performance of *Jump In!* educational response mats used *during* classroom learning, and assessed student outcomes including evaluations of these mats. These mats allow PA to be undertaken simultaneously with content learning, as a complement to course material, as opposed to the predominant view of PA as a break from learning and a supplement to be added on top of the many essential curricular elements competing for teacher and student time during the school day. This view of PA as something extra, rather than as an activity that can coincide with, and indeed contribute to, content learning has enabled PA to be reduced and even removed from many schools despite the high percentage of youth in many countries failing to meet PA recommendations (11, 27, 28).

## Study 1: Focus Groups with Teachers and School Administrators

### Participants and procedure

The following protocol was approved by Colorado State University’s Institutional Review Board (approval ID 13-4120H). Teachers and school administrators were recruited to participate in focus groups about PA in the classrooms via emails that were sent to individual principals (who then distributed them to teachers), as well as flyers that were posted in several schools in Fort Collins, CO, USA. Eleven teachers and one principal participated in these focus groups (8% male). Each focus group was scheduled with a very small group of participants (each of them provided written consent) in order to gather as much data as possible from each individual respondent without requesting that participants devote more than an hour to these discussions. Within each group, a semi-standardized set of questions was designed to be consistent with the needs assessment portion of an Adapted Intervention Mapping approach to public health issues (29). First, participants reported on their beliefs about the benefits of PA for themselves personally and also for students; second, participants reported on their beliefs about how much PA students should get at school, as well as how much PA students actually do get. They were then asked questions about times during the day when students were physically active and what their schools did to support PA. Then, attention turned to whether and how PA could be increased at schools. Participants were asked to describe when and why students were inactive at school, including discussion of specific barriers to PA at school. Finally, teachers were given a description of *Jump In!* educational response mats and then were asked to describe their reactions to this specific tool, the barriers to incorporating such a tool in their school/classroom, and their ideas for how the mats could be incorporated into their teaching.

Paralleling a more recent acceptance and value placed on mixed methodology, qualitative research in public health fields may illuminate phenomena not otherwise captured by purely quantitative methods (30); therefore, focus groups were chosen for the present study for several reasons. First, focus groups are effective for understanding exercise and exercise programs (31, 32), and in particular, in school health exercise research [e.g., Ref. (33)]. In addition, small focus groups were used to facilitate discussion across different participants and different schools in the northern Colorado area. For example, on several occasions, the research team noted that one participant was able to build off of the ideas of the other participant(s). Finally, although the study team was aware of the risk for participants to converge in agreement in a focus group setting, it was deemed of greater importance to approximate venues in which school health issues are discussed and decisions are made (e.g., a school health and wellness committee). Although focus group participants did frequently express agreement about PA in their schools, each focus group with multiple participants also included disagreement about one or more policies or trends at participants’ schools, suggesting that false convergence was not likely occurring.

Six focus groups were conducted with 12 participants; 4 groups had 2 participants, 1 group had 3 participants, and 1 teacher participated alone. Data saturation was achieved with this number of participants. Teachers taught a range of grade levels, although all were elementary school educators. Two teachers taught music, one taught PE, and one taught integrated services; the remaining teachers were primary educators. Teachers and principals represented six schools from in and around Fort Collins, CO, USA; schools represented demographically and geographically (i.e., both urban and rural) different areas.

Data from the focus groups were audio recorded. Consistent with the framework analysis approach to qualitative research (34), the study team used a theme-based approach to analyze results. Upon conclusion of the six focus groups, results were analyzed by: (a) identification of key ideas, (b) classification of typologies, and (c) explanatory analysis with focus on the study’s primary objectives: to better understand school climate around PA, assess school readiness to implement PA initiatives, and explore readiness to use *Jump In!* The research team met on several occasions to reach consensus.

### Results

#### Teacher descriptions of the school climate related to PA

Overall, participants agreed that students were not getting ideal amounts of PA on most school days, although the actual estimates of average daily PA varied from school to school. Most teachers (roughly 75%) estimated that on average students accumulated approximately 30–45 min of PA during the school day, and the majority of this active time was during recess. Participants reported that younger children (kindergarten through third grade) had more opportunities for PA than did older children (in fourth and fifth grade); again, this primarily meant more time spent in recess for the younger children. For instance, one teacher noted that teachers and principals “agree that little kids need time to be active and run around and play but then you reach a certain period like fourth and fifth [grade] and those are the highly, you know, standardized tested grades and it’s like, well, all of the sudden we think they don’t need that … which, you know, obviously I disagree with.”

Several participants reported that there was much more active time on days that students had PE classes, but the frequency of those classes was not consistent among the schools represented by focus group participants, ranging from three times over 4 weeks to approximately every other day. In addition, many teachers spoke very highly of the ability of PE teachers to get all of the children moving for most of the time spent in the class; however, other teachers reported that students spent less than half of the time in PE classes actually being physically active.

Interview participants reported a wide range of percentages (ranging from approximately 10–33%) when asked what percent of students at their schools commuted actively (e.g., walking or bicycling to school), but only teachers at one school described having a lot (but still a minority) of children who commute by bike or by foot. However, the majority of teachers talked at some point in the focus groups about PA occurring frequently during classroom transitions. They reported using many different strategies frequently referred to as “brain breaks” to get children moving for just a few minutes every hour; when asked whether these breaks were used widely by teachers throughout their schools, all teachers reported that they were relatively typical.

Although participants reported that most teachers used brain breaks to encourage PA during the day, they agreed that most students were inactive during classroom learning. However, when these periods of inactivity were problematic, especially for particularly high-energy/fidgety students, most teachers reported using strategies to encourage healthy outlets for energy, rather than punishing hyperactivity (e.g., allowing students to stand in the back of the classroom rather than sit at a desk, allowing students to sit on exercise balls rather than typical chairs).

Overall, participants reported that their school environments and policies were generally supportive of PA. Most typically, this support came in the form of providing playground and sports equipment (the latter was often provided by parent groups or PE teachers). In addition, several teachers reported school-wide initiatives (e.g., bike to school programs, competitions to encourage PA); however, roughly half of the participants reported that there were no official policies to support PA, beyond the district-mandated Wellness Committee at each school. Finally, several schools were moving to celebrating events like birthdays with PA rather than food; when there were policies in place about this issue, teachers reported they were often motivated by food allergies rather than by encouraging PA, *per se*. Taken together, these results suggest that teachers and school administrators believe that students should be more physically active during the school day than they currently are, and that school climates are generally supportive of PA.

### Beliefs about benefits of PA for students

Across the board, focus group participants believed that there were benefits of PA across multiple domains for their students. Most frequently, participants highlighted cognitive and mental health improvements for students. Interestingly, participants did not discuss physical health benefits.

Overwhelmingly, focus group participants highlighted that PA during the school day allowed students to focus more effectively and, likewise, that the absence of PA led to a diminished ability to focus. One participant made a behavioral observation regarding recess and focus. She stated, “I notice a difference on the days they get to go out [for recess]. They are a lot more ready to settle down and work [afterwards].” Another individual made an argument about the importance of PA by observing behavior in the absence of PA. She noted, “What I have observed is when students don’t get the opportunity to exercise, they are not able to focus as well.” Many participants also highlighted the relationship among PA, the brain, and learning. One teacher stated, “[PA] activates a different part of their brain. I can get them thinking with a different part of their brain through PA.” Similarly, another participant stated, “[PA] gets blood into their brain, helps them to retain information.”

In terms of mental health, several participants highlighted the mental health benefits and positive mood changes observed in their students resulting from PA. Broadly, they observed positive effects on student mood. One teacher noted, “They are happier. They need that time to be unstructured and free.” Another individual discussed PA as a protective factor in clinical mental health issues. She stated, “There are certain individuals who are prone to depression, and I know that exercise helps.” Finally, one administrator discussed PA as an intervention strategy. She stated, “We can use PA to change their emotional state – get them out of that stressed state so they have a chance to think.”

### Obstacles to PA in school

Teachers identified several barriers to in-school PA at the state/federal level, at the school level, and at the individual level. Several of these barriers are particularly relevant to a discussion of how to incorporate PA into classroom learning, and are detailed below. However, other barriers would remain even if these new tools were utilized; these include inadequate equipment, money (e.g., only being able to hire PE teachers part-time), and perceptions that students are getting enough PA outside of school time (particularly students who come from wealthier families).

#### 

##### Time (related to federal and state mandates/standards and testing)

Endorsed nearly unanimously across all of the study participants was the barrier of time, and more specifically, lack of time to devote to PA due to federal and state mandates, standards, and testing. One teacher described this barrier and its impact on PA during the school day. When asked about barriers, she stated, “Curriculum. There’s so much. At the elementary school level, they are giving us more and more and more stuff to do. We got rid of afternoon recess because there wasn’t enough [contact time] to meet required times. Those are minutes that are standard.” Another highlighted how pervasive and distressing the issue is: “Teachers are constantly feeling the crunch time. It’s always hanging over your head that you have all of these things to get done.” One participant, in particular, succinctly described the dilemma between classroom instruction time and opportunities for PA. She noted, “When you rob Peter to pay Paul, are you going to take it from writing or take it from movement?”

##### Buy-in from administration/school culture

Another related barrier described by multiple participants was the importance of administrative buy-in for PA initiatives. Several participants described that their participation in programing that included PA was directly related to the emphasis administration did (or did not) place on such activities. One participant highlighted the relationship between PA, administrative emphasis, and lack of teacher time. She described a barrier as “… definitely values of the administration. Buy-in from administration. A lot of what we don’t do is based on the administration in our building. A big initiative across the board won’t happen from teachers – they have so much on their plate.” Furthermore, one PE teacher highlighted his own frustration with bureaucratic and school level barriers. He stated, “Why can’t there be a mandate for PA? We have study after study and it’s proven over and over again that more active kids perform better, but there are blinders put on at the administrative level.” Clearly, the adoption of new PA tools would require buy-in from school administration.

##### Student characteristics

Individual characteristics of students were also discussed including natural inclination toward PA, student choice, and medical concerns, especially respiratory problems. Many teachers discussed that some students seemed “naturally” less inclined toward movement. In addition, some teachers described experiencing a dilemma about whether they should discourage a sedentary, but otherwise desirable activity (e.g., reading at recess) in order to promote PA at recess. Finally, student health and invisible disability were noted barriers. In fact, one participant described it as the most significant barrier to PA during the school day. She stated, “This is the biggest one: Asthma. Students with asthma. So many students on inhalers…” The adoption of tools that incorporate PA into learning would require sensitivity to individual health issues, but could overcome individual differences in inclination toward movement, and tools like *Jump In!* could reduce health risks associated with sitting even for students who used the mats in a low-exertion way (e.g., standing, stepping onto mats), if more vigorous activity was not feasible.

#### Thoughts on *Jump In!*

Focus group participants reported enthusiasm for *Jump In!* as a tool that students would greatly enjoy using and that would contribute to increased activity during the school day without detracting from time spent learning. One teacher thought *Jump In!* could be implemented in her classroom in several ways: “It would be easy: With math … Have them solve a problem and say ‘here’s four choices.’ Reading: We do multiple-choice tests for reading so instead of filling in bubbles we could use that [*Jump In!*] instead. I could see it used for the formal assessments so they are not sitting there for a long time. Informal assessments, too.”

Echoing these sentiments, several teachers indicated that they would find *Jump In!* particularly useful for math lessons and for multiple-choice quizzes in other areas, but that other uses would be possible as well. In fact, despite talking specifically about the mats for only approximately 10 min during each of the focus group sessions, the teachers were able to come up with numerous uses for *Jump In!* in their own classrooms and schools during this brief span of time. One teacher comment reflected these multiple possible uses well: “I think it’s really adaptable,” she said, and described a few of the ways she could use the mats in her classroom: “It would be cool if they used their hands and feet … or if you could hang it on the wall. Or throw a ball on it.” “Or in teams. They have to decide on an answer and run over and click it.”

Teachers thought that given more time and more teachers they could come up with many different ways to use *Jump In!* “I think teachers if they were given enough time to explore it, they would be using it… Once you show it, demonstrate, let them try it, you will come up with a million more ideas…” In addition to the favorable responses to using *Jump In!* with younger students, and the many possible uses for the mats with this age group, one teacher indicated that *Jump In!* could be useful and enjoyable not only for younger students but among high school students as well.

In addition to the positive feedback, focus group participants were asked to discuss what roadblocks they might encounter in using *Jump In!* in their classrooms. The primary potential barrier reported by participants to using *Jump In!* in the classroom was space, both for storing the mats and for using them in the classroom. Many teachers thought that given the amount of space currently occupied by the desks/tables in their classrooms there would not be sufficient space for all of the students to simultaneously place a mat next to or behind their seat without moving furniture around. A minority of teachers also expressed concerns about how much time would be required to get the mats out and ready to use during class. An additional concern included teachers having to modify existing lesson plans to include questions with discrete outcomes (e.g., multiple-choice questions) in order to be compatible with *Jump In!* mats, rather than using open-ended questions as they preferred to use for many topics. Teachers were also worried that the mats may not be sturdy enough to withstand frequent jumping or that there may be breakable parts, given the electronic communication system used to gather response data and communicate it wirelessly to the teacher’s computer/tablet. One participant also wanted to clarify that the mats would not have wires connecting them to one another, as she foresaw such a design could produce a tangled mess of mats and wires in her classroom. Teachers expressed a desire to have different answer choices appear on the mats themselves, rather than A, B, C, D, as the prototypes were marked. Finally, some participants were concerned that students could “cheat” by waiting to answer questions until a classmate had jumped on his or her answer choice, as it would be possible to see the answers classmates were selecting.

### Discussion

Teachers’ perceptions of the benefits of PA align well with existing research on the relationships between PA and mental health, physical health, and cognition (1–6). Although this was a sample of teachers’ from one region in the USA, participants’ perceptions were in line with data from across the world that students do not engage in ideal amounts of PA (11, 12). Consistent with national trends indicating that PA tends to decrease with age (35), the amount of time allocated to recess and PE by school policy was reported to decrease with increasing student age, such that the kindergarteners, first graders, and second graders in some of the schools represented by focus group participants were afforded the opportunity to participate in nearly twice as much daily PA as fifth grade students.

Physical education time has been reduced in many schools in the USA and internationally to allow for more time spent focusing on content-area learning (13, 14), an issue reflected in the focus group results. The focus group participants agreed that, due to current academic demands, the emphasis on standards, and prescribed amounts of time each day devoted to particular curricular components, it would be very difficult to increase levels of student PA at school. Indeed it was clear that adding PA to the school day would not be possible without extending the school day *or integrating PA into content learning*. Some teachers (i.e., the music teachers who participated in the focus group discussions) mentioned already combining movement with content learning in their classrooms. Most other teachers conceived of PA as a “break” from learning, and had not conceived of PA integrated with classroom learning as a possibility. Thus, none of the subject-area primary educators had yet attempted to integrate PA into learning in her classroom, but after viewing the *Jump In!* mats, all were open to the idea of “killing two birds with one stone” by combining PA with classroom instruction. *Jump In!* would allow these teachers to add PA to the school day without taking away from time spent teaching curriculum, and may very well bring about enhancements in student cognition, achievement, and physical and mental health. In addition, the use of such tools would overcome other barriers related to PA, including individual differences in students’ inclinations toward engaging in PA.

The specific feedback about *Jump In!* provided by the focus group participants underscored many strengths and weaknesses of the initial mat design. The set of prototype mats created for this pilot testing did indeed have design limitations (e.g., sturdiness) that will be addressed in future versions of *Jump In!* intended for longer-term use. Some of the mat characteristics that will be enhanced in forthcoming iterations (e.g., the electronic communication between mats and teacher’s computer, the ability to change the images displayed on the surface of the mats from A, B, C, D, to other letters, words, numbers, and pictures) will require a larger budget than was available for prototype design. Encouragingly, nearly all obstacles described by focus group participants can be overcome with modifications to the mats and creativity on the part of teachers and the study team.

As an example of how even seemingly intractable problems may be solved through mat modification and creativity in the classroom, the space constraints described by many participants as a potential roadblock to adopting *Jump In!* could be addressed by reducing the size of the mats, by pairing *Jump In!* mats with standing desks that allow more space behind them for jumping, by placing mats around the periphery of the room, rather than next to desks, or even by imbedding mats into flooring material so that the mats need not to be stored and moved on a regular basis. As one teacher stated in regard to the potential problem of finding classroom space for using *Jump In!*, “Everyone could find a space. We could push desks together to get more floor space. It would just be creative thinking, but it would be manageable.”

In addition, *Jump In!* users concerned that some students could copy the answers provided by their classmates could rearrange the mats in the classroom such that students were facing away from classmates (e.g., in a circle around the periphery of the room), rather than toward them (as in classrooms set up with traditional rows of desks), or the teacher could count down (e.g., 3 … 2… 1… Jump In!) when requesting an answer so that all students jumped simultaneously. This problem could also be addressed technologically, by identifying those responses provided more than X units of time later than a response was requested.

## Study 2: In-Classroom Assessment

### Participants and procedure

The following protocol (13-4120H) was approved by Colorado State University’s Institutional Review Board. To recruit students for the in-classroom assessment, the district wellness coordinator emailed teachers directly to evaluate interest in having classrooms participate in the current study. One teacher responded in enough time to complete the following assessment before the end of the school year. All children in her classroom were sent home with parental consent forms; the teacher tracked consent forms and followed up with parents until the day of the assessment, by which time all but one child’s parents had provided consent. Children provided written assent the day of the assessment.

*Jump In!* was tested in a second-grade classroom in Fort Collins, CO, USA. There were 23 students (21 participated; 52% male) in this inclusive classroom, in which two students were deaf and/or hard of hearing. Authors worked with the teacher of this classroom to help her incorporate *Jump In!* into her existing class plans. In this classroom, students were divided into three groups that rotated through various stations (independent work, game play related to curriculum, and math). Two of these three groups were assigned to use *Jump In!* during the math lesson (*n* = 13); the other group did not use *Jump In!* during this lesson (*n* = 8). The only demographic information available on students was gender; the experimental and control groups (i.e., *Jump In!* users and non-users, respectively) were comparable in terms of gender breakdown, χ^2^(1) = 2.65, *p* = 0.10; the teacher reported that the mean achievement of students was comparable between the experimental and control groups.

At the end of the lesson, each student was individually given a short quiz on the math material covered during their lesson; the number of correct answers was used in analyses. Then, students answered four questions about their experiences during the math lesson: (1) How easy was it for you to pay attention in class today? (four answer choices ranged from 1 = “really easy” to 4 = “really hard”); (2) How interested were you in the math lesson you just did? (four answer choices ranged from 1 = “not at all” to 4 = “extremely”); (3) How much fun was this period of class today? (four answer choices ranged from 1 = “not at all” to 4 = “extremely”); and (4) How alert (or full of energy) did you feel? (four answer choices ranged from 1 = “not at all” to 4 = “extremely”). Happy and sad faces accompanied the answer choices (e.g., for the question about interest, a sad face was printed below “not at all” and a happy face was printed under “extremely”) and the authors as well as the teacher were available to help students if they had trouble understanding the questions.

For analyses, the two groups of students who did use *Jump In!* were combined and compared to the students who did not use the response mats in terms of their performance on the math quiz and their answers to the questions about their experience during the math lesson for the day; these analyses were conducted using *t*-tests to answer research questions about whether the use of the *Jump In!* mats affected: (a) academic performance and (b) student experiences of the math lesson.

### Results

There were no differences in performance on the math quiz, *t*(*18*) = 0.51, *p* = 0.62 (*M*_Jump In_ = 4.08, SD = 0.73; *M*_No Jump In_ = 4.25, SD = 0.71) or in terms of student-reported attention or fun during the day’s lessons, *t*s < |1.54|, *p*s > 0.14. However, students who used *Jump In!* were significantly more interested in the class material, *t*(19) = −2.32, *p* = 0.03 (*M* = 3.69, SD = 0.48) and also rated themselves as significantly more alert, *t*(19) = −2.16, *p* = 0.04 (*M* = 3.85, SD = 0.38) compared to students who did not use *Jump In!* (interest: *M* = 3.13, SD = 0.65; alert: *M* = 3.25, SD = 0.89).

### Discussion

Study 2 demonstrated the feasibility of using *Jump In!* in a primary school classroom in the USA. Those students who used the mats found class material more interesting, reported feeling more alert, and were also observed to be moving around more than their classmates who were not using *Jump In!* These results suggest that there may be benefits to *Jump In!* and that the use of such a tool will not detract from classroom learning. Indeed, the greater interest in curricular material and greater alertness reported by students could lead to improved classroom learning over a longer period of time than we observed in the present study. The enhanced alertness, along with the potential learning gains it could produce over time, is consistent with previous research supporting longitudinal cognitive benefits from engaging in PA [e.g., Ref. (6)]. However, it is important to acknowledge that, although the two groups were comparable in the indices we were able to examine in the current study, it is possible that there were unmeasured differences between the groups that may explain some of these findings.

In the pilot classroom, the mats were placed around semi-circular tables where the students would otherwise sit, and the students stood while reading math questions and jumped to indicate their answers. Students were not observed to “cheat” by looking at other students’ mats, and the scores on the math quiz indicated that the students using *Jump In!* did not perform differently from the control students taking the quiz without the mats. The teacher who welcomed *Jump In!* into her classroom reported that her students enjoyed the mats, and that she did not have to make significant modifications to her lesson plan for the day in order to incorporate these tools. This classroom test suggests that student activity levels can be increased during content learning by using *Jump In!* without increasing teacher burden or impairing short-term student performance or concentration (indeed, interest in course content and alertness was increased among the pilot participants compared to seated control participants). Longer-term testing of *Jump In!* is necessary to determine what effects this tool may have on student learning and health over time.

### General discussion

Despite the many benefits to be gained through PA (1–6), activity time is being reduced in schools in the USA and worldwide in deference to time spent learning content, particularly given the emphasis on standardized testing (13, 14). The present research was undertaken to determine whether incorporating an innovative tool into the classroom to encourage PA *during* content learning would be acceptable and feasible. Taken together, the results of these two studies demonstrated both acceptability and feasibility of this tool. Primary school teachers and administrators reported valuing PA as an asset to learning as well as mental and physical health, and were receptive to the idea of building more PA into the classroom. *Jump In!* was seen by focus group participants as a fun way to incorporate movement into content learning and avoid prolonged periods of sitting [which, in addition to causing students to become “antsy,” as noted by teachers, may itself be detrimental to health; (9)]. Finally, in a second-grade classroom, *Jump In!* was successfully integrated into a typical lesson plan without necessitating significant modification, and was well-received by both teacher and students.

Along with the strengths of including both focus group discussions with teachers and administrators along with an in-class test of *Jump In!*, the present research had limitations as well. First, although it seemed that saturation of themes was achieved across the focus groups, it is possible that a different sample of teachers and administrators may have demonstrated a lower-level of readiness to adopt new PA techniques in their schools and classrooms. Despite potential generalizability concerns, information from the focus groups may be useful for other school personnel and researchers to examine similarity between the schools represented in this study and their own schools of interest. Such between-school comparisons would be helpful to determine if *Jump In!* would be similarly well-received and utilized in other schools and to explore whether populations different from the group investigated here might also enjoy and benefit from *Jump In!*. These participants had agreed to come discuss their school environments, and part of the description they were provided included PA specifically, so it is possible that these teachers knew more about PA in their schools, had a greater interest in the topic, or were stronger proponents of the benefits of PA than teachers who chose not to participate in the focus groups. In addition, it should be reiterated that, although the purpose of the in-class test of *Jump In!* was to demonstrate feasibility, this test occurred in only one classroom, which may not be representative of all classrooms in the tested school or district. Although *Jump In!* demonstrated success objectively and subjectively in the pilot classroom, it is not yet possible to infer that this tool will be easily adopted by all primary teachers and classrooms.

Future research with *Jump In!* will expand testing into more schools and classrooms, and will objectively measure PA among students using accelerometry. Observation of students using *Jump In!* in Study 2 suggests that while, on occasion, PA using these mats could meet the threshold for moderate-to-vigorous PA (e.g., when students answer math questions by jumping many times consecutively), the mats will more frequently produce intermittent, low-intensity activity; therefore, many of the health advantages may be obtained due to reduction of sitting (36, 37) and also due to weight-bearing activities (i.e., standing and jumping) that strengthen bone during formative years (38, 39). Additional testing will reveal how other teachers and other classrooms use *Jump In!*, as it is possible different teachers will elicit different levels of PA from their students when using the mats. Reduction of sitting time is a benefit that is expected to be reaped by all users of *Jump In!*, as it is not anticipated that students would sit on these mats.

As testing of *Jump In!* continues, the mats will be modified to meet the needs of teachers, administrators, and students. A goal of the present line of research is to make *Jump In!* mats commercially available for any school interested in utilizing them, but further research and development will be necessary before this goal becomes a reality. Further research will also investigate the amount and intensity of PA necessary to improve student academic performance, and how *Jump In!* contributes to meeting these critical levels of PA among students of various ages.

Finally, it should be noted that *Jump In!* is just one possible mechanism for combining PA and learning in a way that both can occur simultaneously and synergistically. One of the primary take-away messages from this research is that PA need not serve solely as a break from learning, and is not necessarily incompatible with content-focused classroom lessons. Many teachers and administrators are ready and willing to challenge the *status quo* of PA and content learning as mutually exclusive entities. The present results suggest that the time may be ripe for a qualitative shift in the way in-school PA is conceptualized and undertaken.

## Conflict of Interest Statement

The authors declare that the research was conducted in the absence of any commercial or financial relationships that could be construed as a potential conflict of interest.
